# Hydrogen Repairs LPS-Induced Endothelial Progenitor Cells Injury *via* PI3K/AKT/eNOS Pathway

**DOI:** 10.3389/fphar.2022.894812

**Published:** 2022-05-12

**Authors:** Qingjie Mu, Kaixuan Lv, Jielun Yu, Shangmin Chu, Lichun Zhang, Lingyu Kong, Linlin Zhang, Yan Tian, Xiaopeng Jia, Benhong Liu, Youzhen Wei, Nana Yang

**Affiliations:** ^1^ School of Clinical Medicine, Weifang Medical University, Weifang, China; ^2^ University of Health and Rehabilitation Sciences, Qingdao, China; ^3^ School of Bioscience and Technology, Weifang Medical University, Weifang, China; ^4^ Medical Laboratory Animal Center, Weifang Medical University, Weifang, China; ^5^ Weifang Key Laboratory of Animal Model Research on Cardiovascular and Cerebrovascular Diseases, Weifang, China; ^6^ School of Rehabilitation Medicine, Weifang Medical University, Weifang, China; ^7^ Research Center of Translational Medicine Shanghai East Hospital, Tongji University, Shanghai, China; ^8^ Shandong Qilu Stem Cell Engineering Co., Jinan, China; ^9^ Department of Respiratory, Dongying People’s Hospital, Dongying, China; ^10^ Research Center for Translational Medicine and Key Laboratory of Arrhythmias of the Ministry of Education of China, East Hospital, Tongji University School of Medicine, Shanghai, China

**Keywords:** endothelial progenitor cells, lung injury, hydrogen, PI3K/AKT/eNOS signaling pathway, lipopolysaccharide

## Abstract

Endotoxins and other harmful substances may cause an increase in permeability in endothelial cells (ECs) monolayers, as well as ECs shrinkage and death to induce lung damage. Lipopolysaccharide (LPS) can impair endothelial progenitor cells (EPCs) functions, including proliferation, migration, and tube formation. EPCs can migrate to the damaged area, differentiate into ECs, and participate in vascular repair, which improves pulmonary capillary endothelial dysfunction and maintains the integrity of the endothelial barrier. Hydrogen (H_2_) contributes to the repairment of lung injury and the damage of ECs. We therefore speculate that H_2_ protects the EPCs against LPS-induced damage, and it’s mechanism will be explored. The bone marrow-derived EPCs from ICR Mice were treated with LPS to establish a damaged model. Then EPCs were incubated with H_2_, and treated with PI3K inhibitor LY294002 and endothelial nitric oxide synthase (eNOS) inhibitor L-NAME. MTT assay, transwell assay and tube formation assay were used to detect the proliferation, migration and angiogenesis of EPCs. The expression levels of target proteins were detected by Western blot. Results found that H_2_ repaired EPCs proliferation, migration and tube formation functions damaged by LPS. LY294002 and L-NAME significantly inhibited the repaired effect of H_2_ on LPS-induced dysfunctions of EPCs. H_2_ also restored levels of phosphor-AKT (p-AKT), eNOS and phosphor-eNOS (p-eNOS) suppressed by LPS. LY294002 significantly inhibited the increase of p-AKT and eNOS and p-eNOS expression exposed by H_2_. L-NAME significantly inhibited the increase of eNOS and p-eNOS expression induced by H_2_. H_2_ repairs the dysfunctions of EPCs induced by LPS, which is mediated by PI3K/AKT/eNOS signaling pathway.

## Introduction

Acute lung injury (ALI) and acute respiratory distress syndrome (ARDS) are a series of pulmonary pathological changes arising from a wide variety of lung injuries, which have high morbidity and mortality (Butt et al., 2016), characterized by disruption of endothelial barrier integrity and diffuse lung damage. It can cause an imbalance between coagulation and inflammation to induce inflammation (Frantzeskaki et al., 2017). Besides, macrophages, neutrophils inflammatory cells and their pro-inflammatory products can destroy pulmonary epithelial cells, increase pulmonary microvascular permeability, produce pulmonary edema, damage gas exchange, and lead to respiratory failure (Butt et al., 2016). Therefore, how to maintain the integrity of the endothelial barrier through the regulation of the microenvironment in the inflammatory state is critical to the treatment.

ECs dysfunction and inflammation contribute to the occurrence and development of lung injury. Thus, vascular endothelial repair is an integral part of lung injury repair (Zhao et al., 2020). EPCs are a kind of progenitor cells that can differentiate into vascular ECs (Asahara et al., 1997), which can migrate to the damaged area and differentiate into ECs to participate in angiogenesis or repair, and promote the improvement of endothelial functions (Li et al., 2017). In addition, EPCs can repair the vascular injury and alleviate LPS-induced lung injury, reduce inflammation, and promote bacterial clearance of pneumonia (Mao et al., 2010), which has a broad prospect in the treatment of lung injury.

Hydrogen (H_2_) is an important physiological regulatory factor that has protective effects of anti-oxidation, anti-inflammation, and anti-apoptosis on cells and organs (Huang et al., 2010b). H_2_ can reduce oxidative stress (Song et al., 2011), promote the scavenging of free radicals, and inhibit vascular aging (Iketani et al., 2018). A multicenter, open-label clinical trial showed that hydrogen/oxygen mixed gas inhalation improved disease severity and dyspnea in patients with Coronavirus disease 2019 (COVID-19) (Guan et al., 2020). In addition, H_2_ has an excellent therapeutic effect on inflammation, ischemia-reperfusion injury, diabetes, cancer, atherosclerosis, and other diseases (Li et al., 2013; Lee et al., 2015; Shimada et al., 2016; Li et al., 2019). It can reduce the levels of serum Low-Density Lipoprotein Cholesterol (LDL-C) and apolipoprotein- B (Apo-B), improve the high-density lipoprotein (HDL) functions damaged by dyslipidemia (Song et al., 2013), reduce the formation of neointima after vein transplantation in rats (Sun et al., 2012), and decrease hypertension, angiogenesis imbalance and oxidative stress caused by placental ischemia (Ushida et al., 2016). It also can protect the pulmonary microvessels of mice from the endothelial function damage induced by septicemia, maintain the consistency of pulmonary endothelium (Li Y. et al., 2020), and improve microvascular ECs viability in traumatic brain injury by inhibiting autophagy (Wang Y. et al., 2020). Animal experimental studies have shown that H_2_ inhalation can provide protection in animal models of lung injury caused by mechanical ventilation, sepsis, ischemia-reperfusion, LPS and hyperoxia, seawater infusion, etc. (Ohsawa et al., 2007; Xie et al., 2010; Chen et al., 2015; Diao et al., 2016; Audi et al., 2017). Clinical studies show that the inhalation of H_2_ by pregnant women can also inhibit the LPS-induced apoptosis and oxidative damage of fetal lung cells (Hattori et al., 2015). Previous studies have found that EPCs can repair lung injury induced by LPS (Yang et al., 2019), and H_2_ has the same effect (Hattori et al., 2015). We speculate that H_2_ may have a protective effect on EPCs, and even repair LPS-induced lung injury by improving the functions of EPCs. In this study, the injury model of EPCs was established by LPS treatment, and the cell viability, migration, angiogenic ability, and related protein expression of EPCs were measured. The molecular mechanisms of H_2_ on the functional damage and repair of EPCs induced by LPS were discussed.

## Materials and Methods

### Animals

ICR mice (4 weeks old, males) were obtained from the Cavens Company (Jiang Su, China). All animal experiments were approved by the Animal Experimental Ethics Committee of Weifang Medical University (approval code: 2019SDL108).

### Isolation and Culture of EPCs

MNCs were isolated from the femurs of 4-week-old male ICR mice by density gradient centrifugation using Histopaque 1,083 (Sigma, St. Louis, MO, United States). The isolated MNC were seeded in a 6-well culture plate coated with fibronectin and cultured in EGM-2MV (Endothelial cell basal medium-2, plus FBS, VEGF, R-IGF-1, rhEGF, rhFGF-B, GA-1000, hydrocortisone and ascorbic acid) (Lonza, Basel, Switzerland). After 3 days of culture at 37°C with 5% CO_2_, the culture medium was changed thoroughly with fresh culture medium, and non-adherent cells were removed, and the culture medium was changed every 2 days.

### Characterization of EPCs

MNCs from mice bone marrow were cultured for 5 days, incubated with 50 ug/ml Human Dil-Acetylated Low Density Lipoprotein (Dil-Ac-LDL, FuShen, Shanghai, China) at 37°C for 4 h. Then cells were fixed in 4% paraformaldehyde (PFA), incubated with FITC-labeled Ulex europaeus agglutinin 1 (FITC-UEA-1, FuShen, Shanghai, China) for 1 h. After setting the image acquisition parameters at each wavelength using background control, images were obtained under OLYMPUS, IX71 fluorescence microscope at 400x.

We further detected the expression of surface markers in cells at 10d and 21d. Cells were fixed in 4% PFA, treated with 0.1% Triton X-100 for 10 min. After being blocked with 5% FBS for 1 h at room temperature, the cells were incubated with primary antibodies against CD117 (C-Kit) (eBioscience, San Diego, CA, United States), SCA-1 (Abcam, Cambridge, MA, United States), VEGFR 2 (Abcam, Cambridge, MA, United States), CD31 (Abcam, Cambridge, MA, United States), eNOS (Cell Signaling Technologies, Danvers, MA, United States) overnight at 4°C. After being washed with PBS, EPCs were incubated with secondary antibodies conjugated with Cy3 (Goat anti-mouse cy3, 1:100, Proteintech Group, Chicago, IL, United States; Goat anti-Rat cy3, 1:100, Jackson Immunoresearch Laboratories, West Grove, PA, United States) or FITC (Goat anti-rabbit FITC, 1:100, Proteintech Group, Chicago, IL, United States) for 1 h at 37°C. The immunofluorescence staining was evaluated under a fluorescence microscope (OLYMPUS, IX71, 400x).

### EPCs Treatment

Before the experiment, the cell culture medium was replaced by basic medium (M199 + 5% FBS). Then EPCs were treated with different concentrations (2.5 μg/ml, 5 μg/ml, 10 μg/ml, 20 μg/ml) of LPS (Solarbio, Beijing, China) at different time (24, 48, 72 h) to establish a damaged model. The cell damaged model was treated with H_2_ in different concentrations (20%, 40%, 60%) at different time (24, 48, 72 h) to explore the suitable conditions of H_2_. The concentration of CO_2_ in the H_2_ incubator is 5%, the concentration of O_2_ is 21%, and the concentration of H_2_ is adjusted by N_2_. Finally, EPCs were treated with PI3K inhibitor LY294002 (Sigma-Aldrich, St Louis, MO, United States) (10 μM, 20 μM, 30 μM) or eNOS inhibitor L-NAME (Beyotime, Shanghai, China) (100 μM, 200 μM) to find out the suitable concentration of inhibitors.

### Cell Viability of EPCs

EPCs were evaluated by 3-[4,5-dimethylthiazol-2-yl]-2,5-diphenyltetrazolium bromide (MTT) assay. Cells (100μl, 5×10^4^ cells/mL) were seeded in 96-well plates and cultured for 24 h until adhered to the wall. After different treatments, cells were incubated with MTT (20 μl, 5 mg/ml) for 4 h at 37°C with 5% CO_2_. 200 μl of dimethylsulfoxide (DMSO) was added to each well and shaken for 10 min. The optical density (OD) values at 492 nm were determined using a microplate spectrophotometer (Multiskan GO, Thermo, United States).

### EPCs Migration Assay

EPCs migration was measured by an 8 μm pore 24-well Cell Migration Assay kit (BD Biosciences, San Jose, CA, United States). EPCs suspension (300 μl, 5×10^4^ cells/mL in M199 medium) was added to the upper chamber and cultured according to different groups, and 600 μl EGM-2MV medium was added lower chamber. After different treatments, the chamber was taken out, scrubbed carefully with a cotton swab and rinsed with PBS. The lower cells were fixed and stained with 0.1% crystal violet. Carefully cut off the Polycarbonate film from the base of the upper chamber, seal the film and take a picture under a microscope.

### Tube Formation *in Vitro*

Matrigel (BD Biosciences, San Jose, CA, United States) matrix was dissolved overnight at 4°C. After being placed in a 37°C incubator for 30min, 250 μl Matrigel was added to a 24-well plate. After treatments, 5×10^4^ cells were seeded in the Matrigel-coated plate. After 6 h incubation, Calcein AM was added to staining for 30 min, and the samples were observed and photographed under a fluorescence microscope.

### Western Blot

Total protein was extracted with Radio immunoprecipitation assay (RIPA, Beyotime, Shanghai, China) lysis buffer and quantified with a BCA assay kit (Solarbio, Beijing, China). In total, 25 μg of protein was electrophoresed on a 10% denaturing polyacrylamide gel and transferred onto polyvinylidene difluoride (PVDF) membranes. After being blocked with 7% dried skimmed milk for 3 h at room temperature, the membranes were incubated with primary antibodies against GAPDH (1: 20,000, Proteintech Group, Chicago, IL, United States), AKT (1: 1,000, Proteintech Group, Chicago, IL, United States), p-AKT (1: 2000, Proteintech Group, Chicago, IL, United States), eNOS (1: 1,000, Cell Signaling Technologies, Danvers, MA, United States), p-eNOS (1: 1,000, Cell Signaling Technologies, Danvers, MA, United States) overnight at 4 °C under constant shaking. After washing with TBST buffer (Shandong Sparkjade Biotechnology Co., Ltd.) 4 times (each 5min), the membranes were incubated with the secondary antibodies conjugated to horseradish peroxidase (HRP) for 3 h at room temperature under constant shaking. After washing with TBST buffer for three times (each 5 min), the protein bands on the PVDF membrane were detected using the ECL chemiluminescence detection kit and chemiluminescence gel imaging system (FluorChem Q, ProteinSimple, CA, United States).

### Statistical Analyses

All data are presented as mean ± standard deviation (SD). The data were analyzed using SPSS software (version 26.0, SPSS Inc., Chicago, IL, United States). Differences between three groups or more were analyzed by one-way ANOVA. Values were considered significant at *p* < 0.05.

## Results

### Isolation and Characterization of EPCs

**FIGURE 1:**
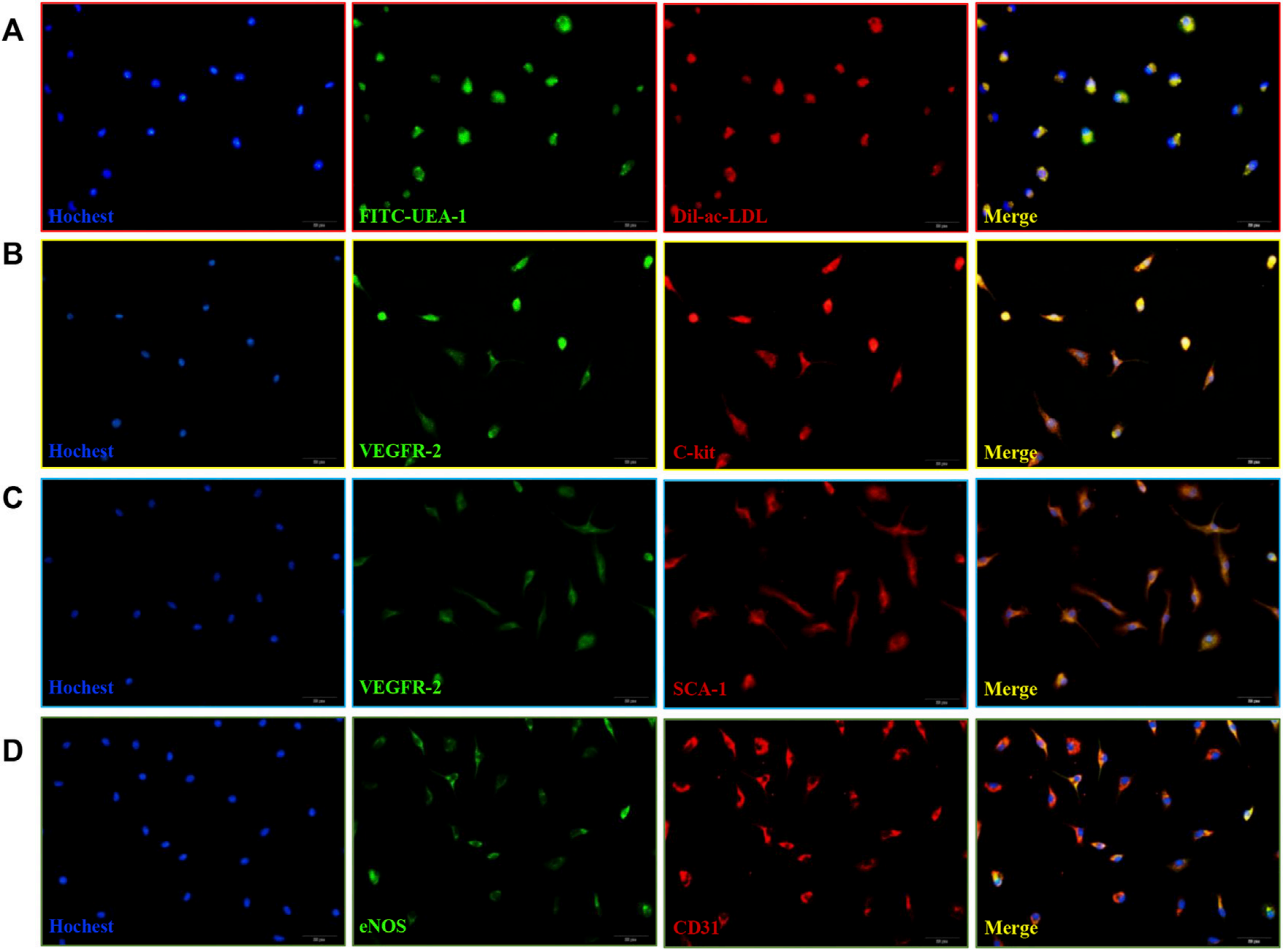
Isolation and characterization of mice bone marrow-derived EPCs. **(A)** DiI-ac-LDL (red) and FITC-UEA-1 (green) could be taken up by EPCs (blue) and merged images of red and green fluorescence. Scale bar = 20 μm (400x). **(B)** Co-expression of VEGFR-2 (green) with C-kit (red) in cells at 10 d. **(C)** Co-expression of VEGFR-2 (green) with SCA-1 (red) in cells at 10 d. **(D)** Co-expression of eNOS (green) with CD31 (red) in cells at 21 days. Scale bar = 20 μm (400x).

Bone marrow mononuclear cells (MNCs) isolated from mouse bone marrow showed cobblestone-like morphology (Supplementary Figure 1). These induced MNCs engulfed Dil-ac-LDL and FITC-UEA-1 (Figure 1A), the differentiation markers of EPCs. Then we examined cell-surface markers of EPCs. After 10 days of culture, VEGFR-2 with C-kit were co-expressed in the isolated cells (Figure 1B), and VEGFR-2 with SCA-1 were also co-expressed. (Figure 1C). After 21 days of culture, the cells expressed both eNOS and CD31. (Figure 1D). Therefore, these induced MNCs were characterized as EPCs and could be used in later experiments.

### LPS Impaired EPCs Functions

**FIGURE 2:**
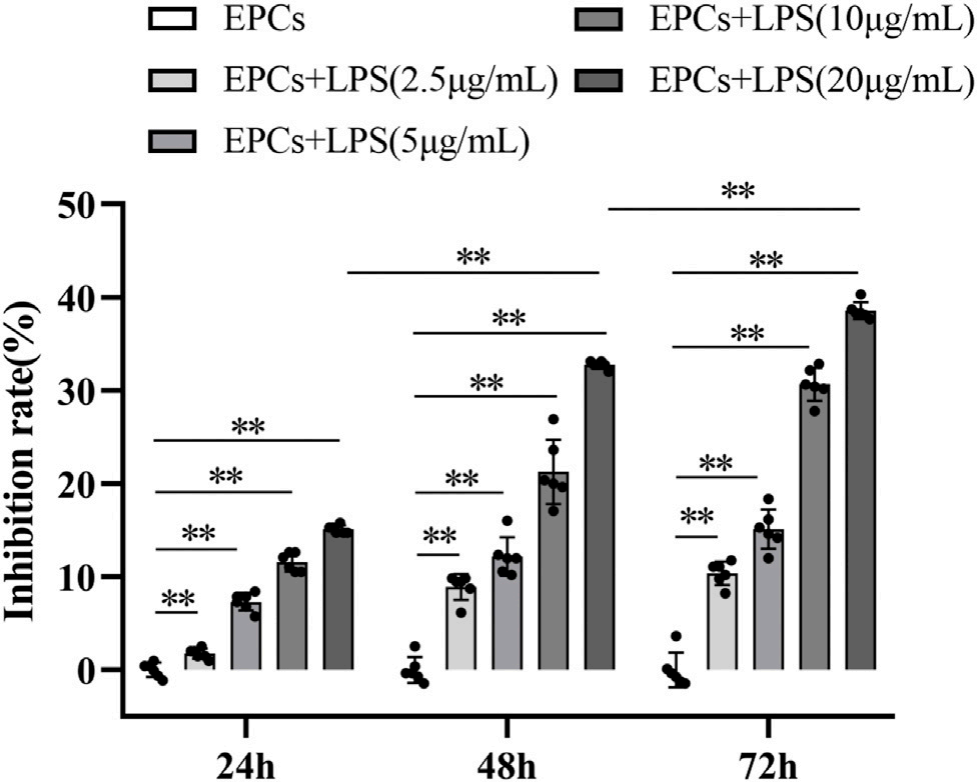
EPCs viability was reduced after being induced by LPS. EPCs were treated with different concentrations (2.5 μg/ml, 5 μg/ml, 10 μg/ml, 20 μg/ml) LPS for different time (24, 48, 72 h). Cell viability was measured by MTT assay. Data are presented as mean ± SD (*n* = 6); **p < 0.01.

**FIGURE 3:**
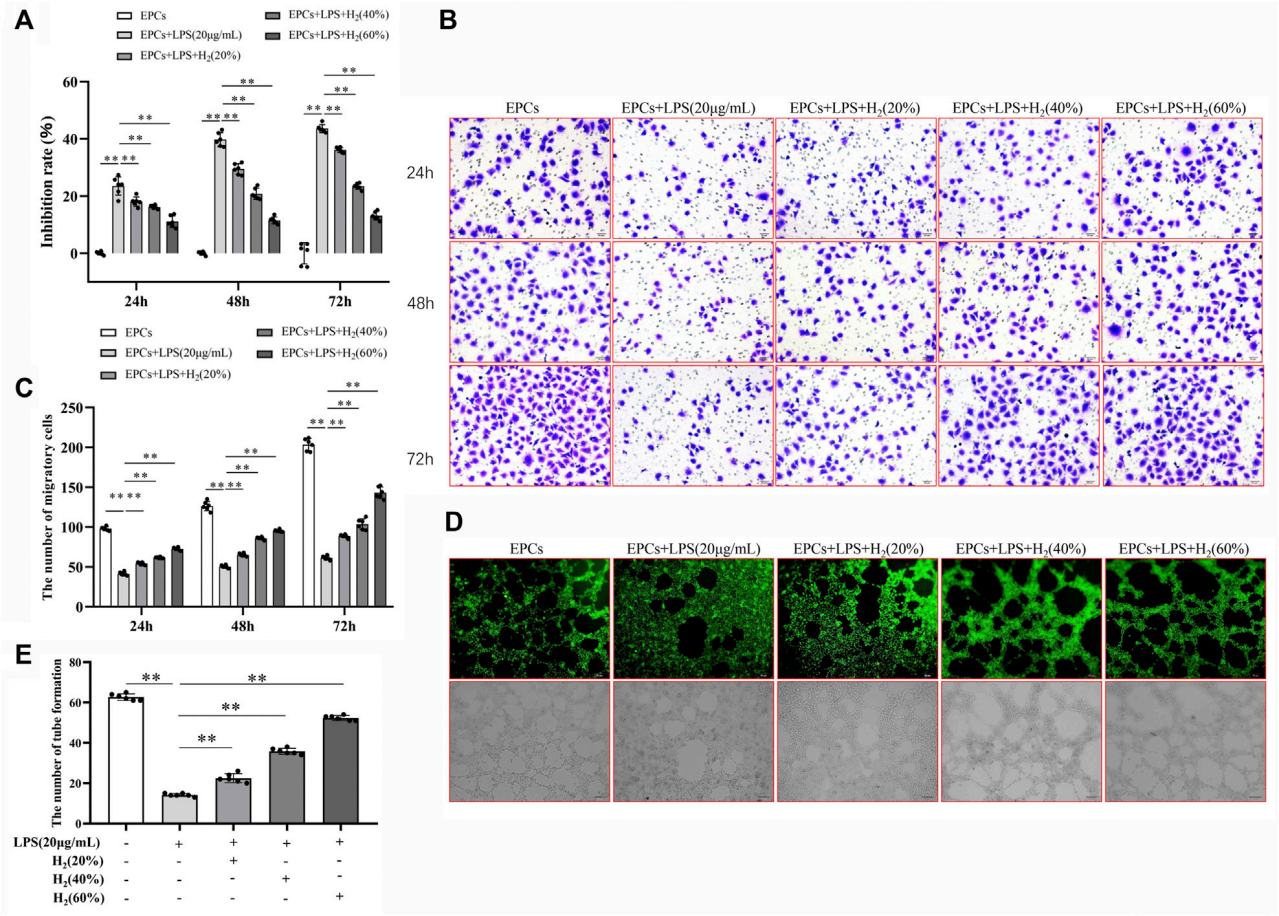
H_2_ alleviated EPCs dysfunctions induced by LPS. EPCs treated with LPS induced EPCs damage, and then EPCs were treated with different concentrations (20%, 40%, 60%) for different time (24, 48, 72 h). The viability of EPCs was assessed by MTT assay **(A)**. EPCs migration was measured by transwell assay **(B,C)**. Tube formation was measured by a Tube formation assay **(D,E)**. Data are presented as mean ± SD (*n* = 6); **p < 0.01.

MTT assay results showed that, compared with the control group, LPS reduced the viability of EPCs in a concentration-dependent and time-dependent manner. We used 20 μg/ml LPS to induce 72 h for the follow-up experiments (Figure 2). In addition, we also found that LPS significantly reduced the ability of migration and tube formation of EPCs (Figures 3B–E).

### H_2_ Alleviated LPS-Induced EPCs Dysfunctions

Compared with the control group, LPS reduced the viability of EPCs. H_2_ significantly improved the LPS-induced reduction in EPCs viability in a concentration-dependent and time-dependent manner, and the effect of 60% concentration of H_2_ on EPCs for 72 h was the most significant (Figure 3A).

Migration assay showed that LPS reduced EPCs migration ability, compared with the control group. H_2_ reversed the LPS-induced changes. H_2_ increased the migration of EPCs in a concentration-dependent and time-dependent manner, and 60% concentration of H_2_ treatment for 72 h has the most significant effect on the repairment of EPCs migration ability (Figure 3 B and C).

The EPCs tube formation was detected with tube formation assay. Compared with the control group, LPS reduces the tube formation of EPCs, which could be reversed by H_2_ treatment in a concentration-dependent manner (Figure 3 D and E).

### LY294002 and L-NAME Inhibited H_2_-Mediated Restoration of EPCs Functions

**FIGURE 4:**
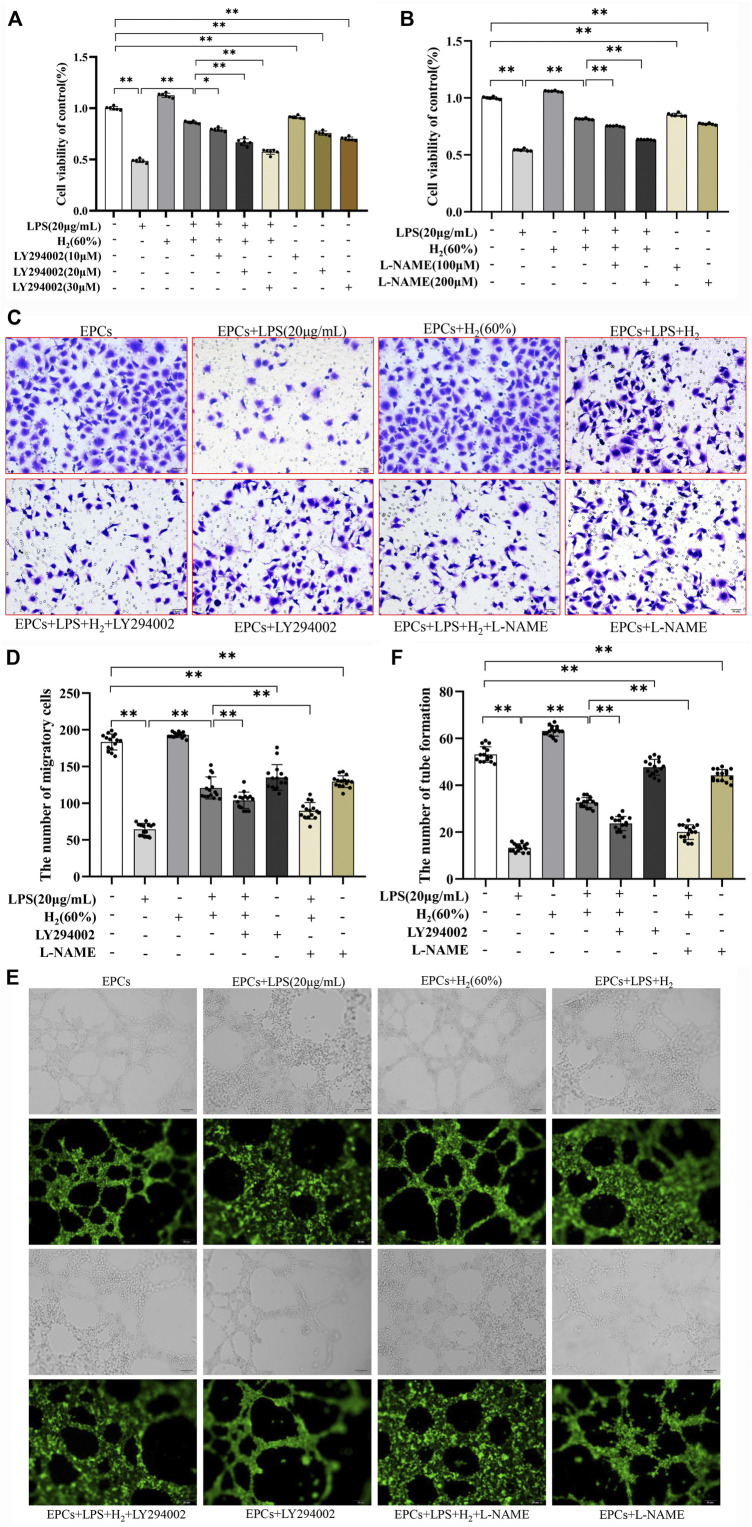
LY294002 and L-NAME inhibited the H_2_-induced repair of EPCs dysfunctions damaged by LPS. EPCs were treated with the PI3K inhibitor LY294002 (10 μM, 20 μM, 30 μM) or the eNOS inhibitor L-NAME (100 μM, 200 μM), incubated with LPS (20 μg/ml) and H_2_ (60%) for 72 h. The viability of EPCs was assessed by MTT assay **(A,B)**, *n* = 6). EPCs migration was measured by transwell assay **(C,D)** *n* = 15. Tube formation was measured by Tube formation assay **(E,F)** *n* = 15. Data are presented as mean ± SD; *p < 0.05 and **p < 0.01.,

The effects of specific inhibitors LY294002 (PI3K inhibitor) and L-NAME (eNOS inhibitor) on EPCs functions were measured to investigate the protective mechanism of H_2_. As shown in Figure 4 A and B, LY294002 (10 μM, 20 μM, 30 μM) and L-NAME (100 μM, 200 μM) inhibited the H_2_-mediated restoration of EPCs viability impaired by LPS. 20 μM LY294002 and 200 μM L-NAME were employed in the subsequent experiments. Figure 4 C-E showed that LY294002 and L-NAME significantly inhibited the H_2_-mediated restoration of EPCs migration and tube formation ability damaged by LPS.

### H_2_ Restored the PI3K/AKT/eNOS Pathway Inhibited by LPS

**FIGURE 5:**
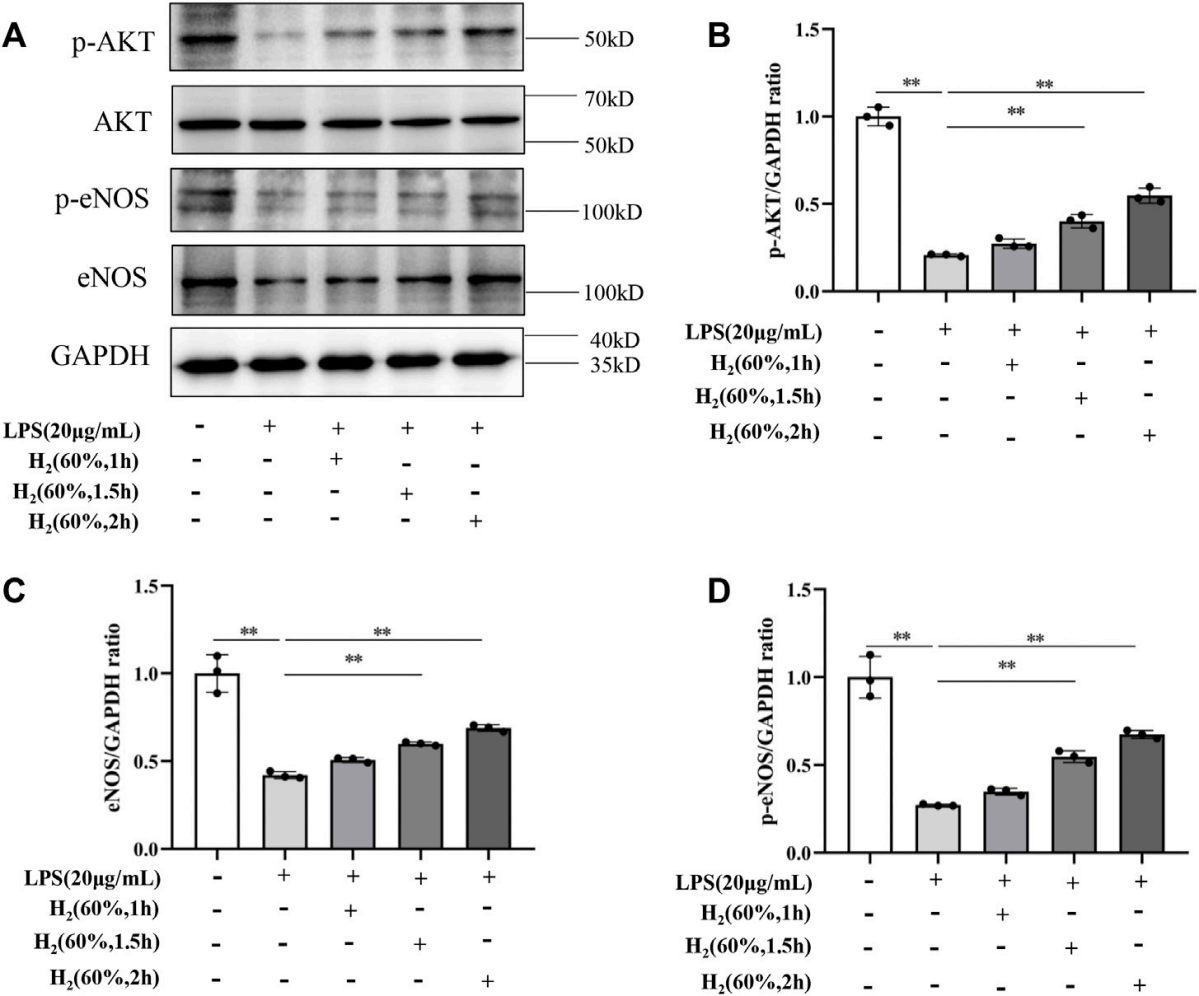
**(A)** Representative western blot images. H_2_ up-regulated the levels of p-AKT **(B)**, eNOS **(C)**, and p-eNOS **(D)** in EPCs at different time points. Cells were treated with LPS (20 μg/ml) and H_2_ (60%) for 0, 1, 1.5 and 2 h, respectively. Western blotting was used to detect the expression levels of target protein. Data are presented as mean ± SD; **p < 0.01.

**FIGURE 6:**
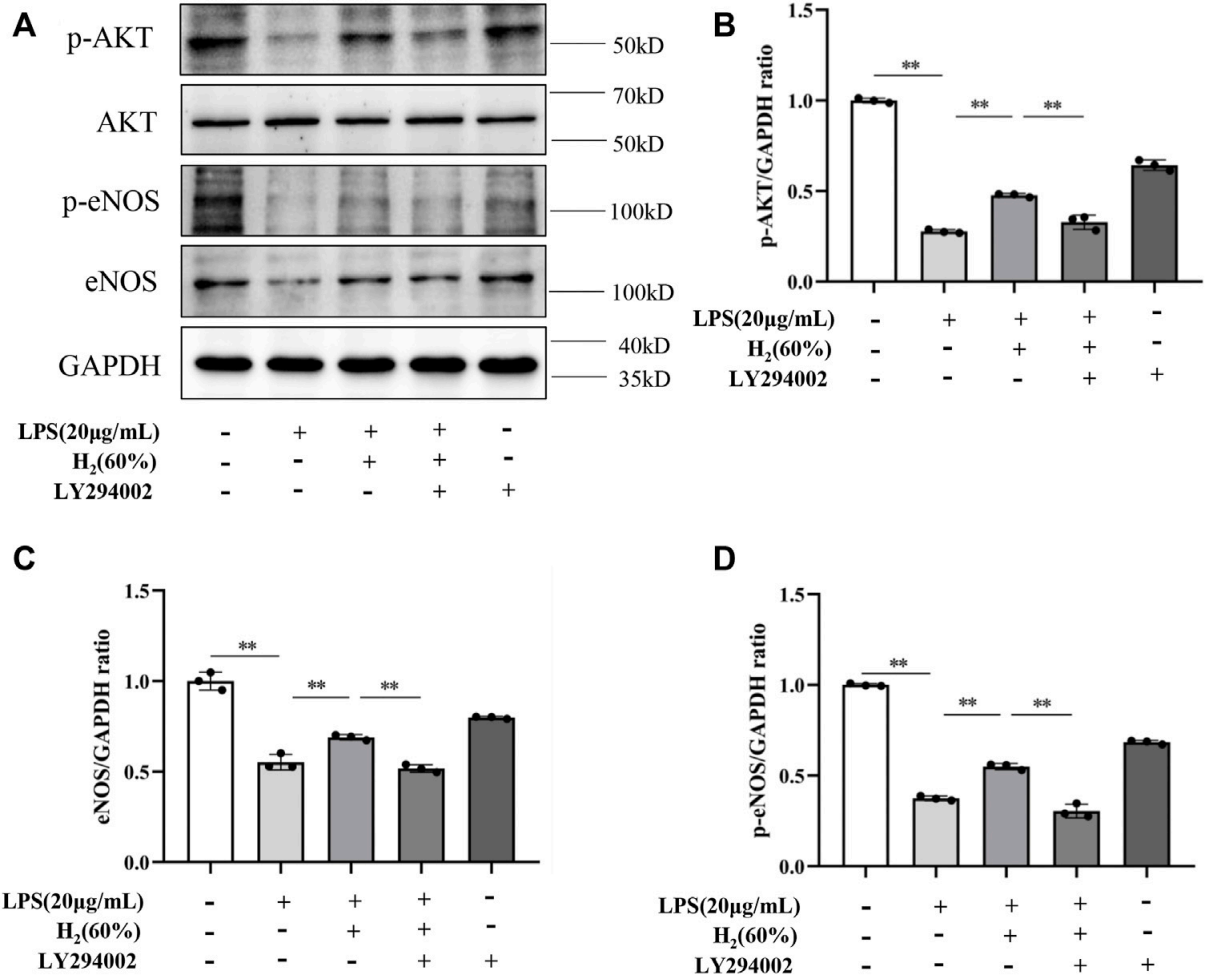
**(A)** Representative western blot images. LY294002 inhibited the increase of p-AKT **(B)**, eNOS **(C)** and p-eNOS **(D)** expression induced by H_2_ (60%). Cells were treated with LPS (20 μg/ml), H_2_ (60%) and LY294002 (20 μM) for 2 h, respectively. Western blotting was used to detect the expression levels of target protein. Data are presented as mean ± SD; **p < 0.01.

**FIGURE 7:**
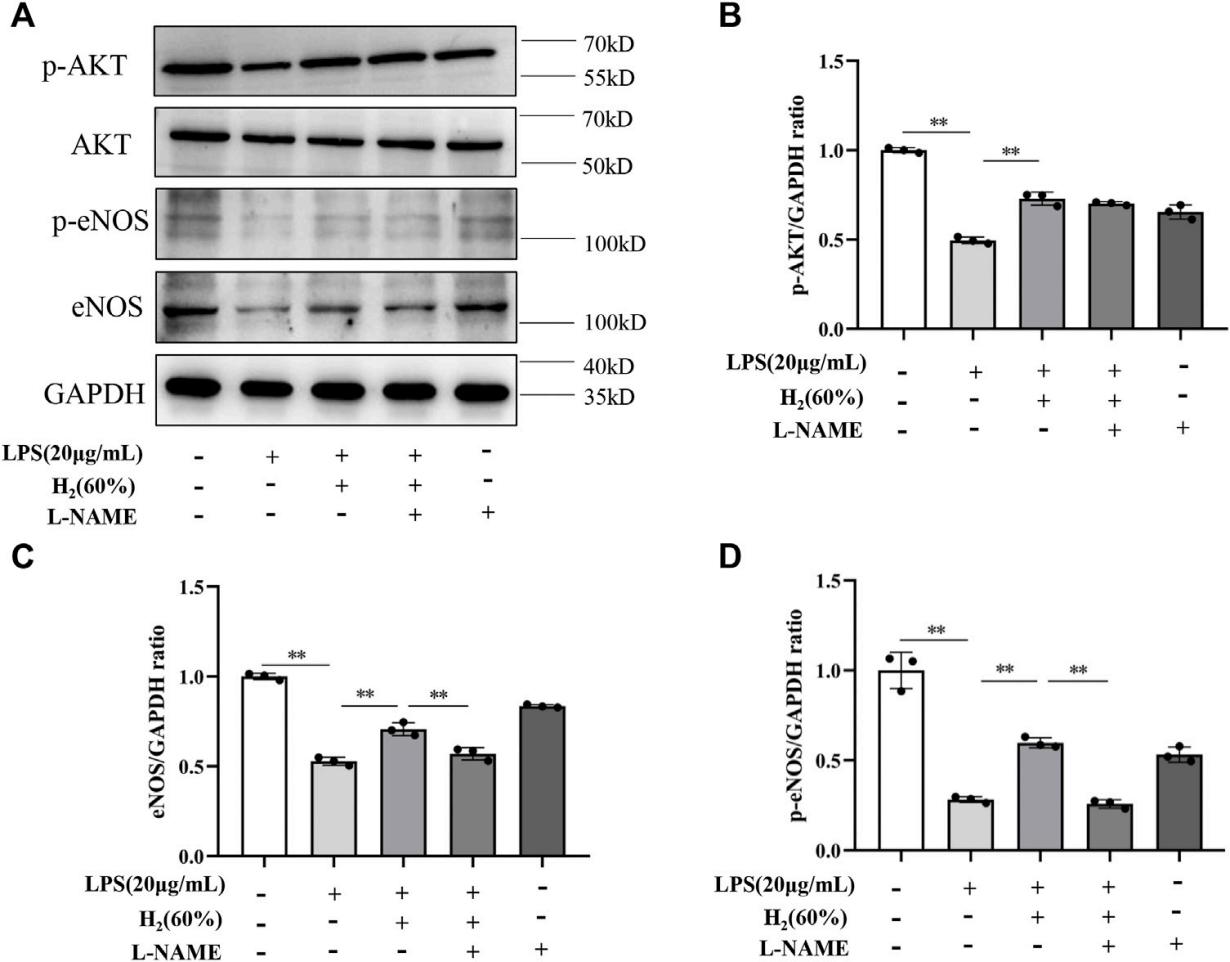
**(A)** Representative western blot images. L-NAME inhibited the increase of eNOS **(C)** and p-eNOS **(D)** expression induced by H_2_ (60%), and did not affect p-AKT levels **(B)**. Cells were treated with LPS (20 μg/ml), H_2_ (60%) and L-NAME (200 μM) for 2 h, respectively. Western blotting was used to detect the expression levels of target protein. Data are presented as mean ± SD; **p < 0.01.

We further investigated the protein levels of AKT, p-AKT, eNOS and p-eNOS to clarify the relationship between H_2_ and PI3K/AKT/eNOS signaling pathway. As shown in Figure 5, LPS (20 μg/ml) decreased the protein levels of p-AKT, eNOS, p-eNOS in EPCs. H_2_ restored these protein levels in a time-dependent manner, and H_2_ treatment for 2 h was employed in the subsequent experiments. As shown in Figure 6, LY294002 significantly inhibited the increased levels of p-AKT, eNOS, p-eNOS induced by 60% H_2_. Figure 7 showed that L-NAME significantly inhibited the increased levels of eNOS and p-eNOS induced by 60% H_2_, however, there was no effect of L-NAME on the expression levels of p-AKT.

## Discussion

EPCs were first initially discovered in 1997 by Asahara et al. EPCs are defined as a cell population capable of differentiating into mature ECs and have vasculogenic potential, which contributes to vasculogenic, wound healing (Asahara et al., 1997) and repair of ischemic tissue damage (Fan et al., 2014). As a potential therapeutic agent, EPCs have attracted attention for a variety of diseases including cerebral ischemia (Zhou et al., 2021), diabetes (Wang K. et al., 2020), ALI etc. (Asahara et al., 1997). However, due to differences in the isolation, amplification and identification of EPCs, as well as controversy over therapeutic function, the further development and clinical application of EPCs were limited. In this study, we successfully isolated the mouse bone marrow-derived EPCs for subsequent research of the repair mechanism of H_2_ on EPCs dysfunctions induced by LPS. This study is the first report of the effect of H_2_ on EPCs.

H_2_ is an odorless, colorless, tasteless, and insoluble multifunctional medical gas. It can cross the cellular membranes and has the functions of anti-oxidation, anti-inflammatory and anti-apoptosis (Hong et al., 2010). In 1975, Dole et al. found that high-pressure hydrogen gas has an antioxidant effect and can inhibit the growth of tumors in mice (Dole et al., 1975). However, the study was not taken seriously because of the limitations of the experiment and the difficulty of reproducibility. In recent years, research on H_2_ in the medical field has gradually widened. Clinical studies have found that H_2_ plays a therapeutic role in diseases which related to the respiratory system (Guan et al., 2020), nervous system, cardiovascular (Camara et al., 2019), digestive system (Eryilmaz et al., 2020), reproductive system, urinary system (Gokalp et al., 2017), and metabolic exercise (Qiu et al., 2020). It is recommended to inhale a mixture of H_2_ and oxygen (O_2_) (33.3% O_2_ and 66.6% H_2_) in the “Clinical Guidance for COVID-19 Pneumonia Diagnosis and Treatment (Trial Version 7)” issued by the China National Health Commission. The recommendation recognized the importance of H_2_ in contemporary medical gas research.

In this study, LPS (20 μg/ml) significantly reduced the proliferation, migration, and tube formation of EPCs. This is consistent with Yu et al. research, which found that LPS (10 μg/ml) impaired the viability, migration, adhesion abilities, and tube formation of late EPCs (Yu et al., 2017). Compared with the control group, the proliferation and adhesion activities of bone marrow-derived EPCs were impaired in LPS (100 ng/ml) induced group in dose and time dependence (Li et al., 2014). Our previous study also demonstrated that LPS (30 μg/ml) inhibited the proliferation, migration, and tube formation of EPCs (Yang et al., 2019). Furthermore, western blot results showed that LPS reduced the expression levels of p-AKT, eNOS, and p-eNOS in EPCs. LPS had no effect on AKT expression. Our previous research proved that LPS (30 μg/ml) decreases the levels of p-AKT, eNOS, and p-eNOS in EPCs (Yang et al., 2019). Yang et al. found that LPS (10 μg/ml) reduced the expression of p-eNOS in human pulmonary microvascular endothelial cells (HPMECs) (Yang et al., 2018). Liu et al. found that p-AKT was markedly suppressed in H9c2 cells after treatment with LPS (1 μg/ml) for 24 h (Liu et al., 2021). The effect of LPS on AKT expression in out study was consistent with the study of Li et al. and Zhan et al. Li et al. showed that there was no significant change in AKT expression of LPS-induced (20 μg/ml) mouse microvascular ECs (Li H. et al., 2020). Zhan et al. reported that the AKT level of HPMECs did not change markedly under LPS (1 mg/L) induction (Zhan et al., 2020). In addition, Wang et al. found that p-AKT protein expression was significantly increased in LPS-induced (100 ng/ml) rat microvascular ECs (Wang et al., 2013). Fan et al. found that LPS (100 ng/ml) stimulation significantly increased the phosphorylation of both AKT and eNOS in HPMECs (Fan et al., 2020). Taken together, we speculate that the inconsistent study results may be due to the differences in LPS concentration. In addition to toxic effects, LPS also has extensive biological activity. Low doses of LPS have immune-activating effects (Morris and Li, 2012) (Lin et al., 2009). Therefore, there are differences in the functional gene expression and cell functions under different concentrations LPS treatment. However, the relevant mechanism still needs further confirmation.

The effectiveness of molecular hydrogen has been proven in the prevention and treatment of many diseases. H_2_ had a protective effect on the rat model of ALI (Jiang et al., 2013; Audi et al., 2017) by significantly improving lung endothelial permeability, reducing cell apoptosis and histopathological changes (Diao et al., 2016), and preventing LPS-induced pulmonary ECs dysfunction (Li Y. et al., 2020). Fu et al. demonstrated that hydrogen-rich saline has a protective effect on LPS-induced ALI by regulating cell apoptosis and inhibiting endothelial dysfunction (Fu et al., 2020). Inhalation was the most direct way to administer molecular hydrogen (Huang et al., 2010a; Kawamura et al., 2010). At the same time, molecular hydrogen could be dissolved in physiological saline to make hydrogen-rich water for intravenous injection, or it could be taken orally (Cardinal et al., 2010; He et al., 2013). A hydrogen incubator was used in this study. It could simulate the *in vivo* environment after inhaling hydrogen, which was more conducive to our research. H_2_ has a protective effect on the ECs barrier, mechanism of which has not been fully elucidated, and the effect of H_2_ on EPCs has not been reported. Our study indicated that H_2_ attenuated the dysfunctions of EPCs induced by LPS, improved EPCs proliferation, migration, tube formation, and restored the expression levels of p-AKT, eNOS, p-eNOS. Recent studies revealed that H_2_ could inhibit the expression of inflammatory factors, reduce sepsis-induced endothelial damage and inflammation, improve endothelial dysfunction (Cardinal et al., 2010; He et al., 2013).

Previous studies showed that the PI3K/AKT/eNOS pathway is involved in the changes of the LPS-induced ECs barrier function (Zheng et al., 2018), but whether it is involved in the effect of H_2_ on EPCs repairment remains unclear. The results of the present study discovered the unique molecular basis for H_2_ to inhibit LPS-induced EPCs dysfunctions. After inhibiting the PI3K/AKT/eNOS signaling pathway by LY294002 and L-NAME, the H_2_-mediated restoration of EPCs functions was partially prevented. The western blot results demonstrated that H_2_ up-regulated p-AKT, eNOS and p-eNOS levels were inhibited by LPS. LY294002 significantly inhibited the increase of p-AKT, eNOS and p-eNOS induced by 60% H_2_. L-NAME significantly inhibited the increase of eNOS and p-eNOS induced by 60% H_2_, and had no effect on p-AKT levels. PI3K/AKT/eNOS activation plays a crucial role in our study. PI3K/AKT signaling pathway mediates a variety of pathophysiological processes and involves multiple important cellular activities, such as cell proliferation, apoptosis and autophagy (Ravikumar et al., 2010; Liby and Sporn, 2012; Saiprasad et al., 2014). Additionally, numerous studies show that the PI3K/AKT signaling pathway plays a vital role in the process of EPCs proliferation, migration, homing and tube formation (Everaert et al., 2010; Wang et al., 2011). Our previous results demonstrate that Rev-D4F mediates restoration of EPCs functions by PI3K/AKT/eNOS signaling pathway (Yang et al., 2019). The PI3K/AKT/eNOS pathway was involved in restoring the dysfunctions of EPCs in diabetic mice (Cao et al., 2017). Yu et al. suggested that the proliferation, migration and survival of EPCs impaired by LDL cholesterol via the PI3K/AKT signaling pathway (Yu et al., 2010).

**FIGURE 8:**
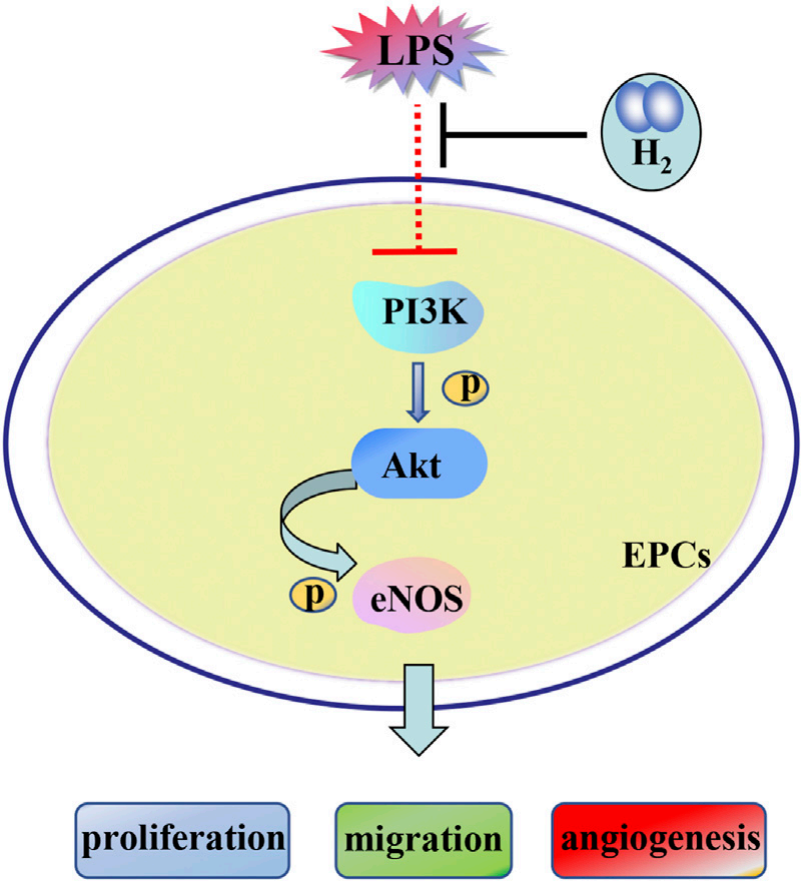
H_2_-mediated restoration of EPCs dysfunctions is mediated by the PI3K/AKT/eNOS pathway.

In summary, we concluded that the PI3K/AKT/eNOS signaling pathway was contributed to H_2_ repairment of EPCs dysfunctions induced by LPS (Figure 8).

## Conclusion

Our results showed that H_2_ reversed the LPS-induced EPCs dysfunctions. Moreover, H_2_ restored the LPS-attenuated levels of p-AKT, eNOS and p-eNOS. Therefore, this study proves that H_2_-mediated restoration of EPCs dysfunctions is mediated by the PI3K/AKT/eNOS pathway.

## Data Availability

The raw data supporting the conclusion of this article will be made available by the authors, without undue reservation.
