# Supplementary figures and images for: Hydrogen Repairs LPS-Induced Endothelial Progenitor Cells Injury via PI3K/AKT/eNOS Pathway
Source: Front Pharmacol. 2022 May 12;13:894812. doi: 10.3389/fphar.2022.894812 (PMC9133378; doi:10.3389/fphar.2022.894812)

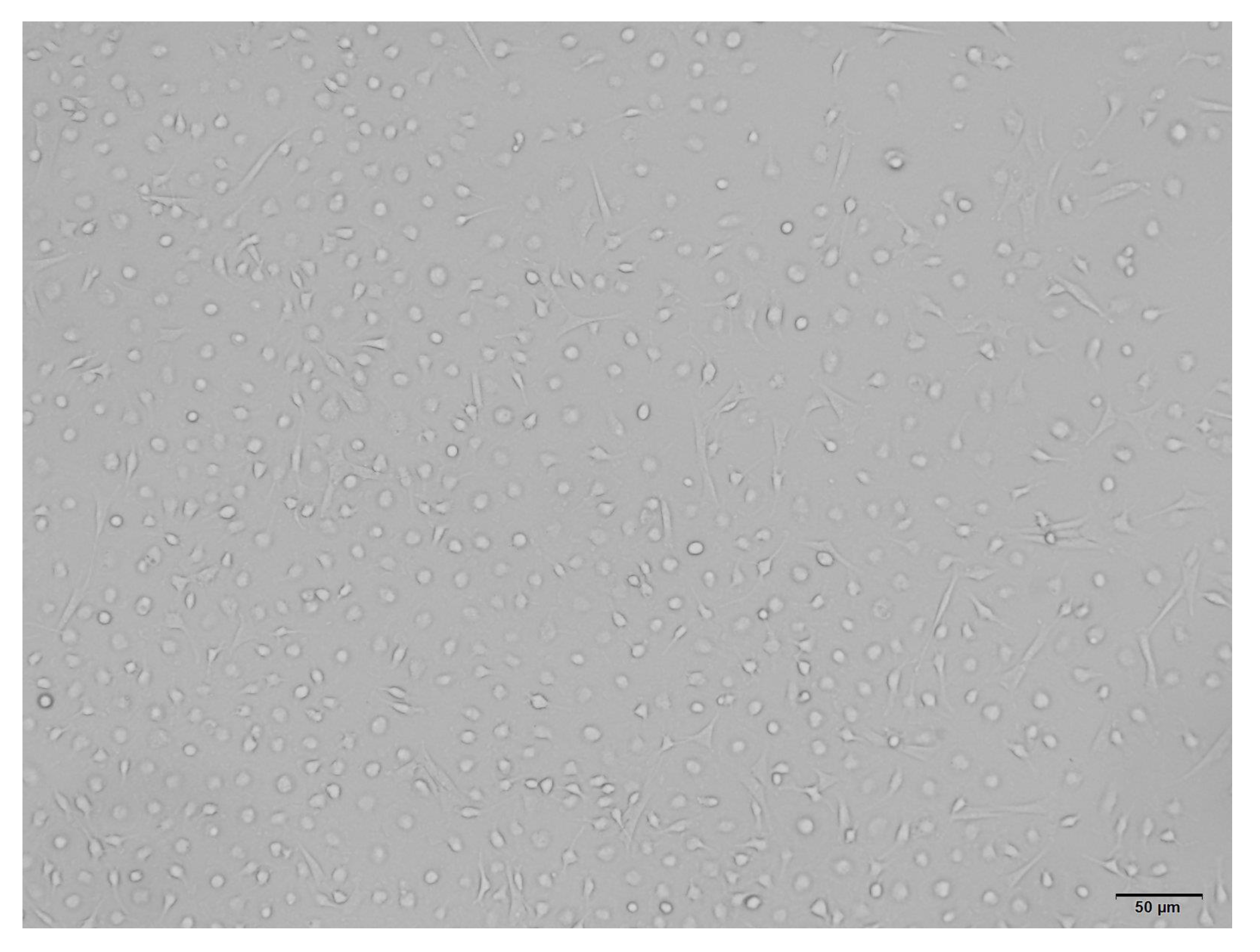

Supplement: Supplementary file 1 [file Image1.TIF]
